# Presenteeism and noise perception at work: a cross-sectional study using association analysis

**DOI:** 10.1590/1516-3180.2021.0792.R2.07042022

**Published:** 2022-08-29

**Authors:** Renata da Silva Cardoso Rocha Tavares, Luiz Felipe Silva, Jorge Muniz

**Affiliations:** I PhD. Researcher, Department of Production, Universidade Estadual Paulista (UNESP), Guaratinguetá (SP), Brazil.; II PhD. Lecturer, Natural Resources Institute (Department), Universidade Federal de Itajubá (UNIFEI), Itajubá (MG), Brazil.; III PhD. Associate Professor, Department of Production, Universidade Estadual Paulista (UNESP), Guaratinguetá (SP), Brazil.

**Keywords:** Presenteeism, Noise, occupational, Occupational health, Sickness presence, On-the-job illness, Noise at work, Noise exposure, Occupational health risks

## Abstract

**BACKGROUND:**

Presenteeism refers to the presence of a worker at work with reduced performance due to illness, and it is a common public health problem. Exposure to noise during production processes brings risk to workers’ health.

**OBJECTIVE:**

To analyze the health profile of workers in the automotive industry and identify the association between noise perception and presenteeism among workers in the Brazilian automotive industry.

**DESIGN AND SETTING:**

This was a cross-sectional study utilizing a case study design analyzing the automotive industry in the interior of São Paulo state, Brazil.

**METHODS:**

This study included 306 workers using the Presenteeism Work Limitations Questionnaire protocol. Multiple logistic regression was used for data analysis.

**RESULTS:**

Male workers with reports of headache, tension, and limited well-being at work, having perceived that noise exposure interferes with productivity, showed a positive association with the occurrence of presenteeism. Physical demand had the highest score in terms of interfering with the presenteeism index.

**CONCLUSION:**

Workers’ perceptions of noise were associated with presenteeism.

## INTRODUCTION

The quality of work performance and the health of a worker impact productivity, and they have been important subjects in the work world,^1^ and they influence a company's costs, reputation, and competitiveness.

Traditionally, studies have analyzed the financial impact of absenteeism in production,^2–4^ however, they point out that presenteeism today can become the absenteeism of tomorrow.^2^ Some argue that the effects of presenteeism on an individual's future health are unknown, but it is recognized that it cannot be underestimated as a cause of potential health risks.^5,6^ Furthermore, presenteeism can accentuate existing health problems and increase the risk of illness and absence from work, as well as negatively affect workability.^7^

Presenteeism can be defined as being present at work, but being limited with respect to some aspects of the development of work due to a health problem.^8–12^ Presenteeism has been conceptualized as a measurable loss of worker performance due to health problems in the workplace, which contributes to the economic costs related to lost productivity,^13^ and in some countries, represents the main occupational health problem.^6^ A higher prevalence of presenteeism has been observed nationwide among young workers with higher education levels experiencing and reporting pain, impaired sleep, and stress, presenting an overall negative perspective on life.^14^

Treated as a public health concern, presenteeism can act as a risk to the health and safety of others, as the decision to go to work while being affected by an infectious disease, for example, represents a situation that entails possibilities of health disorders caused by exposure to viruses and bacteria, particularly when one interacts with more vulnerable people.^15^ Presenteeism is therefore a poorly-studied phenomenon that has assumed greater magnitude in the field of health.^7^

A large-scale study in the United States, which included a four-year review of presenteeism data from a healthcare system, showed that chronic back pain, mental illness, general anxiety, severe migraines or headaches, neck pain, and depression were the causes of the greatest loss of estimated daily productivity and that allergies and headaches had the highest annual cost.^16^

With regard to well-being, workers exposed to occupational noise had more work-related illnesses and tended to continue carrying out the activity despite inadequate health conditions, thus signifying an association with the occurrence of presenteeism.^17^

Data from a recent survey conducted in Australia relating to hearing loss from noise exposure to productivity and quality of life showed that under current levels of occupational noise exposure in Australia, it is estimated that over 80,000 male and 31,000 female workers would develop occupational noise-induced hearing loss over 10 years of such exposure. Following this cohort to age 65, the estimated loss of productivity-adjusted life-years was 135,561, with a projected loss of 21.3 billion Australian dollars.

Studies on corporate health management have tended to study the impact of health on productivity and the financial impact of this relationship,^11,18–20^ however, practically all the research that covers the subject of presenteeism was developed in capitalist countries.

From this perspective, the influence of self-reported noise exposure in the workplace on productivity, and its association with presenteeism have been under-investigated. This paper seeks to explore this gap by unveiling how certain health-related factors interfere with productivity, including addressing the workers' perception of exposure to occupational noise in an automotive components company.

## OBJECTIVE

To analyze the health profile of workers in the automotive industry and investigate the association between presenteeism and noise in the work environment, as expressed by their own perceptions.

## METHODS

### Ethical considerations

The study was approved under No. 123854/2016 on (March 14, 2017) by the Research Ethics Committee São Paulo State University (Universidade Estadual Paulista, UNESP), Institute of Science and Technology, Campus São José dos Campos (SP), Brazil. All participants signed an informed consent form before participating in the study.

### Procedures

This cross-sectional study was inspired by the research of Merrill et al.,^11^ whose design is defined as a case study with a quantitative approach.

The studied company operates exclusively in the metallurgy sector, producing and marketing components for the automotive sector, located in the eastern region of the state of São Paulo, with 3,600 employees at the time of the field research carried out in June 2018.

### Data collection instruments

To obtain data related to presenteeism, the Work Limitations Questionnaire (WLQ)^21^ was used, validated for the language spoken in Brazil (Portuguese),^22^ and developed by the researchers to collect sociodemographic and health data. The WLQ has four domains of work limitation, comprising 25 items: (1) time management (five items), (2) physical demand (six items), (3) mental-interpersonal demand (nine items), and (4) production demand (five items). After evaluating the four domains of work limitations, it was possible to set a WLQ index.

The sociodemographic and health questionnaire was divided into four sections to obtain data on noise exposure and hearing, and also on workers' health in the last 12 months from the date of application of the questionnaire.

### Sampling

Based on the 20% prevalence of presenteeism observed in the study by LeCheminant and Merril^11^ in which the highest quintile (20% worst) was considered, a sample size value of 320 workers was found, considering the 95% confidence interval and absolute precision of five percentage points.^23^ We used the convenience sampling method, with the questionnaires being handed out to workers who moved through the company's leisure area.

The sample exclusion criteria were as follows: situations in which the worker answered all the questions as “not applicable” were excluded from the analysis, per the recommendation of the WLQ protocol—outsourced employees, employees who were at the company for less than one month of work, and questionnaires in which the subject failed to answer all the questions.

### Data analysis

The collected data were organized using Microsoft Excel software version 2017 (Microsoft Office, Redmond, United States), and then analyzed using the statistical program Epi-info 3.5.2 (created by the Centers for Disease Control and Prevention (Atlanta, United States).

Association analysis controlling for confounding variables was performed using unconditional multiple logistic regression. The dependent variable was defined as “presenteeism,” and categorized according to the WLQ index obtained as a cutoff point, wherein the value “1” signified greater or equal, and “0” for less than that defined by the cutoff. The explanatory variables (independent) used to build the model, based on the literature review, identified in association with the occurrence of presenteeism, were: factors related to noise and hearing (presence of tinnitus, complaints of difficulty in hearing, frequency of exposure to noise, perception of noise as an interference in work productivity), sociodemographic factors (age, sex, education, marital status), professional characteristics (position, work shift, work sector, working time), health issues and “style” of life (classification of one's health in general, quality of life and sleep, alcohol consumption (more than twice a week), smoking, presence of high blood pressure, diabetes, stress, anxiety, depression, asthma, heart problems, allergy, back pain, headache, and body mass index (BMI), and aspects related to work (feeling of well-being at work, tension/stress at work).

Univariate analyses were conducted to build a multiple model, with the entry into the modeling process having a P-value < of 0.20 based on the likelihood ratio test. The stepwise forward methodology was used to define the most appropriate model, in which the variables were included in descending order of significance, and non-significant variables that could interfere with the adjustment of the model were excluded, analyzing the variations in the values of the odds ratio (OR) values, confidence interval (95% CI) and the significance levels of the models. The significant variables in the final model were also verified by the previous test, allowing for the permanence of variables with p being less than or equal to 0.05.^24^

## RESULTS

Of the 320 questionnaires applied, 14 (4.4%) were discarded for the following reasons: some were incomplete, and others filled out the entire protocol with the option “not applicable.” Exclusion from the analysis in this situation followed the stipulations of the protocol, resulting in 306 valid questionnaires, that is, 95.6% of the planned sample.

### Sample characterization

The sociodemographic profile of the 306 workers who responded to the questionnaire, whose mean age was 33.6 ± 9.7 years, is shown in Table 1. Most of the sample consisted of male workers, married workers, and those who had completed high school.

**Table 1:** Distribution of sociodemographic data of a sample of workers in an automotive industry, 2018 (n = 306)

Variables		Mean ± SD
**Age**		33.6 ± 9.7 years
**Seniority**		9.4 ± 8.1 years
**Variables**		**n (%)**
**Gender**	Female	24 (7.8)
	Male	282 (92.2)
**Marital status**	Single	113 (36.9)
	Married	185 (60.5)
	Divorced	7 (2.3)
	Widower	1 (0.3)
**Education**	Incomplete Elementary School	2 (0.7)
	Complete Elementary School	11 (3.6)
	Incomplete High School	2 (0.7)
	Complete High School	199 (65.0)
	Incomplete Higher School	31 (10.1)
	Complete Higher School	61 (19.9)

SD = standard deviation.

Table 2 presents the data on the health conditions of the workers in the sample. In general, a high percentage of the workers in the sample declared their health and quality of life to be good, with good sleep patterns. Furthermore, they reported proportions that did not exceed 5% in terms of behaviors harmful to health, such as smoking and the use of alcoholic beverages more than twice a week. The distribution in relation to physical activity too was among the strata. The findings regarding hearing and other risk factors and their occurrence in the last 12 months are shown in Table 3.

**Table 2:** Distribution of basic data on health and lifestyle in a sample of workers, in an automotive industry, 2018 (n = 306)

Variables	Stratum	n (%)
**BMI** (kg/m^2^)	Underweight (0-18.5)	5 (1.7)
	Healthy (18.6-24.9)	133 (44.3)
	Excess weight (25-29.9)	121 (40.3)
	Obesity (level I, II, III) (≥ 30)	41 (13.7)
**General health**	Very bad/ bad	1 (0.3)
	Regular	30 (9.8)
	Good/very good	275 (89.9)
**Quality of life**	Very bad/bad	9 (2.9)
	Regular	51 (16.7)
	Good/very good	246 (80.4)
**Sleep quality**	Very bad/bad	34 (11.1)
	Regular	83 (27.1)
	Good/very good	189 (61.8)
**Alcohol intake**	Never	108 (35.3)
	Rarely	101 (33.0)
	Up to twice a week	87 (28.4)
	Three times or more in the week	10 (3.3)
**Physical activity**	Never	69 (22.8)
	Rarely	68 (22.4)
	Up to twice a week	76 (25.1)
	Three times or more in the week	90 (29.7)
**Smoking**	No	293 (95.7)
	Yes	13 (4.3)

BMI = body mass index.

**Table 3:** Distribution of variables related to health and work conditions of the last 12 months (n = 306)

Variables	Stratum	n (%)
**Allergy**	Never	190 (62.1)
	Sometimes	64 (20.9)
	Frequent	33 (10.8)
	Very common	19 (6.2)
**Back pain**	Never	140 (45.9)
	Sometimes	118 (38.7)
	Frequent	30 (9.8)
	Very common	17 (5.6)
**Headache**	Never	138 (45.1)
	Sometimes	131 (42.8)
	Frequent	23 (7.5)
	Very common	14 (4.6)
**Occupational noise exposure**	Never	31 (10.1)
	Sometimes	28 (9.2)
	Frequent	63 (20.6)
	Very common	184 (60.1)
**Noise interference in productivity**	Does not interfere	150 (49.0)
	Interferes little	68 (22.2)
	Partially interferes	59 (19.3)
	Totally interferes	29 (9.5)
**Well-being at work**	Very bad/bad	4 (1.3)
	Regular	55 (18.0)
	Good/very good	247 (80.7)
**Tension at work**	Indifferent/not very tense	177 (57.8)
	Partially tense	68 (22.2)
	Tense/very tense	61 (20.0)

### WLQ protocol

From the perspective of the scope of the WLQ protocol, the demand results are as follows:

- Regarding the time management factor, there was no significant attribution of value to this item; that is, in relation to the last two weeks, the individual was limited to performing their tasks at work with regard to the time management limiter (7.46%).
- The physical demand factor had the highest score (16.09%), indicating that, across all demands, physical state had the greatest influence on the productivity of the analyzed sample.
- Regarding mental and interpersonal demands, interference in and subsequent loss of productivity was estimated at 8.43%.
- For production demand, a value of 9.33% of lost productivity was obtained.

According to the WLQ calculation protocol, a score above five indicates an estimated 4.9% decrease in productivity. Considering that the findings from the WLQ Index were equal to 2.74 in the sample studied, it can be said that the loss of productivity due to presenteeism is negligible or non-existent. It is noteworthy that the total presenteeism score of the WLQ measures the impact of chronic diseases and their treatment on the performance and productivity of workers. This finding corroborates the low prevalence observed in the sample in relation to chronic diseases.

### Association between variables and WLQ Index

The univariate analysis, indicating the significant explanatory variables associated with presenteeism (WLQ Index), is shown in Table 4.

**Table 4:** Significant explanatory variables associated with the occurrence of presenteeism, through univariate analysis, with the respective values of OR and P-values in the automotive industry, 2018 (n = 306)

Variables			Total Score WLQ - means	
			OR	P value
**Sociodemographic data**				
	Gender	2.97	0.012	
	Education		0.6535	0.110
	Work shift		0.53	0.0097
**Health data**				
	General health		3.18	0.004
	Quality of life		2.14	0.011
	Sleep		1.71	0.034
	Alcohol intake		0.6623	0.132
	Stress		3.53	0.013
	Anxiety		2.1645	0.111
	Depression		3.1245	0.140
	Asthma		2.9361	0.115
	Smoker		0.4016	0.198
**Noise and hearing data**				
	Bad hearing		2.49	0.030
**Health and work conditions of the last 12 months**				
	Headache		7.84	< 0.001
	Noise interferes in productivity	3.06	< 0.001	
	Tension at work	4.33	< 0.001	
	Well-being at work	3.00	< 0.001	
	Allergy	2.38	0.006	
	Back pain	2.32	0.010	
	Occupational noise exposure	1.9207	0.052	

OR = odds ratio; WLQ = work limitations questionnaire.

Thus, when analyzing Table 4, it is observed that workers who reported headaches had a 7.8 times chance of presenteeism than those who did not report any health problems. With regard to tension at work, the value index was 4.3 times the chance of presenteeism compared to those who did not declare it.

Analyzing the WLQ Index in the final model adjusted by multiple regression, there lay an association with significant explanatory variables: headache, noise interference at work, tension at work, well-being at work, and being a male (Table 5).

**Table 5:** Final model adjusted for explanatory variables associated with the occurrence of presenteeism, with the respective values of OR, CI (95%), and P-values, in an automotive industry, 2018 (n = 306)

Variables	OR	CI (95%)		P value
		Lower	Upper	
Male	2.80	1.06	7.36	0.037
Headache	5.12	2.19	11.97	< 0.001
Noise interference in productivity	1.92	1.05	3.50	0.033
Tension at work	3.07	1.73	5.45	< 0.001
Feeling of well-being at work less than expected	1.96	1.00	3.82	0.047

OR = odds ratio; CI = confidence interval.

## DISCUSSION

The study revealed a predominance of male workers, a characteristic found across research in the metal-mechanical sector, especially in terms of production,^25,26^ and other studies^27,28^ that limited the sample to male workers. The age of workers, represented by the average value, is consistent with other studies in the sector.^25–28^ In terms of education, the findings are in line with those observed by Picoloto and Silveira.^29^

Regarding health data, alcohol consumption among workers was similar to that of national standards, according to the National Health Survey of the Brazilian Institute of Geography and Statistics (Instituto Brasileiro de Geografia e Estatística, IBGE),^30^ while physical activity was higher and smoking was much lower than that from the same source.

Because it was a relatively young sample, with regard to self-reported chronic degenerative diseases, the prevalence of hypertension and diabetes was lower than the national reference used, but similar when compared to the occurrence of cardiovascular diseases.

With respect to asthma as well as depression, in comparison with the country-level estimate, the prevalence values were also lower in the studied sample. Taking into account the values shown in Table 3 and comparing it with data from IBGE,^30^ the value of chronic back or back problems is close to the findings in this study.

In relation to the prevalence of frequent attacks of respiratory allergy among the workers in the sample, the study by Collins et al.^31^ discusses that allergies, including respiratory allergy, are one of the most common factors among chronic diseases that impact presenteeism.

Also, as demonstrated in Table 3, it can be observed that 80.7% reported exposure to noise with a certain frequency in the work environment, and 28.8% reported this noise having some impact on their working capability. We expound that such findings are related to the fact that the researched company has a vast array of old machinery, thereby resulting in a noisier work environment.

Of the sample, 12.8% reported having tinnitus, and 8.5% reported not being able to hear well. Tinnitus is the perception of a sound that originates in the ears or head, without the presence of an external source, thus decreasing an individual's auditory sensitivity, with numerous possibilities of origin.^32^ Another study demonstrated an association between the presence of tinnitus and hearing loss due to occupational noise.^33^

Exposure to intense noise for prolonged periods leads to auditory (hearing loss) and non-hearing effects, such as changes in the neurological system, circulatory system, digestive system, endocrine system, immune system, and psyche.^34^ Thus, the effects of noise exposure can compromise several other organs, devices, and functions of the body, such as changes in the cardiovascular and gastrointestinal systems, endocrine system, and muscle and mood changes. It is also associated with the occurrence of stress, irritability, dizziness, and a greater probability of accidents at work.^35^

### Association between variables and WLQ index

Pain with regard to presenteeism has been studied for a long time. Headaches appear to be a factor that interferes with presenteeism (5.12 times the chance compared to those without pain), thereby corroborating the findings of several studies.^16,31,36,37^

Regarding the demand for well-being, the study by Muckenhuber et al.^41^ mentions well-being as a factor strongly associated with presenteeism. For such a variable, there is also an option to use the individual well-being index (IWB) protocol to explore this association.^39^

Corroborating the findings of this study, two studies involving workers in the health sector found a significant association between male workers and presenteeism.^40,41^

Regarding tension in the work environment, another significant variable, several studies have revealed the association between the occurrence of presenteeism and the presence of stress at work.^42–46^

With regard to noise exposure, a study conducted on railway workers focused on its association with well-being. The authors found that workers exposed to noise reported greater illness but were more likely to continue working anyway; that is, presenteeism.^17^

The association between noise perception and presenteeism was also identified in a study by Tavares et al.,^47^ who evaluated workers in the mining sector.

It is well-established in the literature that excessive noise exposure can cause noise-induced hearing loss (NIHL). There is scientific evidence proving that workers with NIHL have a relative reduction of 1.9% in productivity at work (presenteeism) when compared to workers without hearing loss.^48^ Published data from a survey conducted in Australia concluded that reducing noise exposure at work would substantially reduce the economic burden of occupational noise-induced hearing loss, also related to the loss of quality-adjusted life years (QALY) and productivity-adjusted life years (PALYs).^49^

It is noteworthy that the “body weight” factor, represented by the BMI, was not significant. This finding is in line with the study conducted by Bustillos, Vargas, and Gomero-Cuadra,^50^ which examined work productivity among adults with varied BMI using Canadian Community Health Survey population-based data, between 2009 and 2010. The results reflected that, in relation to normal BMI, the chances of absenteeism were higher for those in obesity class III. Presenteeism was weakly associated with all obesity categories (Class I obesity).

The self-reported variable “asthma” did not present a significant association with presenteeism in this study, contrary to the findings of Sadatsafavi et al.,^51^ who found that presenteeism was more responsive to asthma control than absenteeism.

The manifestation of presenteeism in organizations, such as working while sick, can be interpreted as organizational citizenship behavior and a sign of commitment and loyalty to the company, or even because labor relations are unequal and authoritarian. There also exists criticism against a few organizations discouraging employees from going to work when they are sick.^52^

This research founded physical demand as the most significant cause of presenteeism, in contrast to the findings of Merrill et al.,^53^ stating that demand related to the work environment was higher. In this study, we did not find a relationship between presenteeism and age, marital status, education, work area (sector), being overweight, or smoking, in contrast to the findings of the previous authors' research.

These research findings contribute to the improvement of the strategies used by companies for productivity and health management, that is, indicators that demonstrate the impact of the analyzed factors and, with this, allow for improvements in prospects regarding workers' quality of life, which fall in line with increased productivity. The novelty of this article lies in the fact that no other research has sought to analyze the relationship between presenteeism and the noise variable, while also using a research instrument validated internationally as well as in the Portuguese language. In a search conducted in the Web of Science database (2013–2021), using the keywords “presenteeism” and “noise,” and without any search filter, four articles were identified, of which three mentioned the relationship between presenteeism and noise,^54–56^ but did not aim to analyze or quantify this relationship.

As for the relationship between presenteeism and noise, there have been findings in which the factors most frequently listed as having adverse health effects among 53 educators from daycare centers in Frankfurt were noise and stress;^54^ researchers investigated the environmental and health characteristics related to productivity in a complex of government buildings and highlighted that noise was one of the factors frequently reported by the participants, along with the prevalence of presenteeism in this sample.^55^ In the research by Schell et al.,56 presenteeism and noise were listed, among other factors, to identify occupational health characteristics in a sample of 1961 workers with a wide variety of professional skills and occupational tasks. However, the studies demonstrate limitations as they do not measure the impact and/or relationship between presenteeism and the noise variable. This study contributes to this gap in knowledge by quantifying the impact of the perception of noise exposure and presenteeism score, thus meeting the indicated needs.

Data on presenteeism is scarce in the organizational environment, and consequently, strategies aimed at reducing presenteeism are minimal. The focus of corporate health programs remains limited to reducing absenteeism. Thus, there exist no efforts aimed at managing health as an active asset, reducing it to merely managing expenses and profits.

Studies that seek to investigate the impact of productivity related to chronic and occasional pain, levels of noise exposure by sector, sleep quality, job satisfaction, personal factors such as family-related factors, financial factors, organizational culture, and even promotion projects will add, synergistically, toward improvements in the effective management of workers in the long term promotion of corporate health, or even the sustainability of human capital.

This study encourages discussions in the field of worker and corporate health, involving issues of presenteeism and absenteeism in the organizational sphere, as well as encouraging researchers to discuss issues of this nature to promote improvements in this field and expand on the realm of extant knowledge. It is also necessary for future research to address the methodological importance of differentiating between the act and impact of presenteeism,^57^ across different fields of work.

## CONCLUSIONS

This study analyzed the association between the occurrence of presenteeism and exposure to occupational noise based on the perception of the workers of a company in the metallurgical (automotive) segment in Brazil.

It was concluded that perceived occupational noise is associated with presenteeism. Among other variables, in relation to health, the most relevant were headaches and the perception of well-being at work. Tension in the work environment and the perception of noise exposure were significant factors with regard to presenteeism.

Since the mean age in the sample consisted of younger workers, it is inferred that, for this reason, chronic diseases do not manifest themselves as important variables in the loss of productivity.
